# Impact of Electrospun Piezoelectric Core–Shell PVDFhfp/PDMS Mesh on Tenogenic and Inflammatory Gene Expression in Human Adipose-Derived Stem Cells: Comparison of Static Cultivation with Uniaxial Cyclic Tensile Stretching

**DOI:** 10.3390/bioengineering9010021

**Published:** 2022-01-08

**Authors:** Walter Baumgartner, Petra Wolint, Silvan Hofmann, Cléa Nüesch, Maurizio Calcagni, Marzia Brunelli, Johanna Buschmann

**Affiliations:** 1 Division of Plastic and Hand Surgery, University Hospital Zurich, Rämistrasse 100, CH-8091 Zurich, Switzerland; walter-baumgartner@bluewin.ch (W.B.); Petra.Wolint@usz.ch (P.W.); Silvan.Hofmann@usz.ch (S.H.); Clea.Nueesch@usz.ch (C.N.); Maurizio.Calcagni@usz.ch (M.C.); 2 2EMPA, CH-9014 St. Gallen, Switzerland; brunelli.marzia@gmail.com

**Keywords:** poly(vinylidene fluoride-co-hexafluoropropylene), polydimethylsiloxane, adipose-derived stem cells, coaxial electrospinning, bioreactor, elastic modulus, residual strain

## Abstract

Specific microenvironments can trigger stem cell tenogenic differentiation, such as specific substrates or dynamic cell cultivation. Electrospun meshes composed by core–shell fibers (random or aligned; PDMS core; piezoelectric PVDFhfp shell) were fabricated by coaxial electrospinning. Elastic modulus and residual strain were assessed. Human ASCs were seeded on such scaffolds either under static conditions for 1 week or with subsequent 10% dynamic stretching for 10,800 cycles (1 Hz, 3 h), assessing load elongation curves in a Bose^®^ bioreactor system. Gene expression for tenogenic expression, extracellular matrix, remodeling, pro-fibrotic and inflammatory marker genes were assessed (PCR). For cell-seeded meshes, the E modulus increased from 14 ± 3.8 MPa to 31 ± 17 MPa within 3 h, which was not observed for cell-free meshes. Random fibers resulted in higher tenogenic commitment than aligned fibers. Dynamic cultivation significantly enhanced pro-inflammatory markers. Compared to ASCs in culture flasks, ASCs on random meshes under static cultivation showed a significant upregulation of *Mohawk, Tenascin-C* and *Tenomodulin*. The tenogenic commitment expressed by human ASCs in contact with random PVDFhfp/PDMS paves the way for using this novel highly elastic material as an implant to be wrapped around a lacerated tendon, envisioned as a functional anti-adhesion membrane.

## 1. Introduction

Major characteristics of tendon tissue are the absence of vessels, very few tendon cells with low metabolic turnover and a poor healing capacity [1]. After rupture, tendons recover with conservative healing or surgical repair. However, particularly for flexor tendon surgical repair, a major issue consists in the adhesion of the new forming matrix to the surrounding tissue [2], leading to a compromised range of motion and joint stiffness [2]. Besides this, the development of scar tissue may result in reduced mechanical stability. Consequently, re-ruptures occur after excessive load in 7–15% of cases [3]. With regard to tendon repair optimization, stem cell therapy is becoming increasingly popular [4,5] due to the ability of stem cells to differentiate toward a tendon-like phenotype known as tenogenic lineage when appropriately stimulated through external cues. For example, tenogenic differentiation of adipose-derived stem cells (ASCs) was achieved by supplementing growth factors to the culture medium, such as growth and differentiation factor-5 (GDF-5) [6].

In order to prevent adhesion to the surrounding tissue, conventionally sutured tendons can be wrapped with biological and artificial membranes [7], acting as a physical barrier besides influencing cell growth and differentiation. Moreover, due to controlled drug delivery, surface topography and/or specific chemical characteristics, membranes’ interaction with stem cells were shown to reduce scar formation [8] and to modulate inflammatory events [9] which are reported to have a high impact on proper tenogenesis [10].

Piezoelectric materials have been shown to affect stem cell differentiation [11,12], as cells are able to sense the electrical stimuli provided by cyclic stretching/compression of the piezoelectric substrate. For example, electrical stimulation via piezoelectric materials led to a differentiation in neural stem cells [11], embryonic stem cells towards cardiomyocytes [13] and osteogenesis in mesenchymal stem cells [14,15].

Beyond the classic ceramic materials which are well known for their piezoelectric properties [12,16], polyvinylidene fluoride (PVDF)-based materials were shown to be suitable polymeric substrates for electrical stimulation [17]. Compared to ceramics, polymers have the further advantage to allow simultaneous electrical and mechanical stimulation due to their adequate mechanical elasticity. Using this approach, cells seeded onto piezoelectric polymeric membranes undergo a double stimulation. The first stimulation regards the electric stimulation due to the piezoelectric effect of the material under dynamic loading, and the second concerns the physical stretching of the membrane [18]. The impact of mechanical stimulation on stem cells’ commitment is indeed well known [19,20]. As tendons are physiologically exposed to stretching during force transmission, dynamic stretching of tissue engineered constructs in bioreactors can support a tenogenic commitment [21,22,23,24]. Among the key parameters, applied strain, duration and frequency of stretching were shown to play a fundamental role during stem cell commitment towards the tendon phenotype, while depressing osteoblast, chondrocyte and adipocyte phenotypes [25].

Among piezoelectric polymers, PVDFhfp (poly(vinylidene fluoride-co-hexafluoropropylene) provides support for tissue engineering applications [26,27]. Indeed, PVDFhfp is biocompatible, bioinert and finds already wide application in the development of cardiovascular devices, such as coronary stents [28]. Moreover, the polymer can be easily electrospun to extracellular matrix-mimicking scaffold meshes [29]. Stretched nanofibers of PVDFhfp have been reported to act beneficially though their piezoelectric effect: the mechanical-to-electrical conversion of stretched and aligned nanofibers outperformed the random sample by more than 10 times in terms of enhanced energy harvesting capability [30]. Using coaxial electrospinning, fibers featuring a core embedding a different material can be fabricated, further tailoring the mechanical properties of the meshes. PVDFhfp in combination with other polymers like polydimethylsiloxane (PDMS) was shown to provide core–shell electrospun fibers whose elasticity can be tuned and adapted to different technical purposes [15,29,31]. For example, such a membrane has been shown to help separating water from diesel fuel [31] or to be used as a flexible nano-generator to harvest sustainable energy [15].

The advantage of developing fibers with a PDMS core and a PVDFhfp shell resides in the possibility to obtain highly elastic fibers whose interface with the external environment is controlled by a very stable and biocompatible material. Indeed, the hydrophobic nature of PVDFhfp hinders proper cell attachment, which enlarges the value of a good physical barrier function to prevent tendon adhesion and optimize the gliding capacity in the tendon sheath of intrasynovial tendons [32,33,34,35].

In this study, a novel coaxially electrospun core–shell meshes, composed of either aligned or random oriented PVDFhfp/PDMS microfibers, were seeded with human ASCs without further chemical supplementation; no tenogenic induction medium. Furthermore, ASC-seeded PVDFhfp/PDMS random fiber meshes were cultivated for one week under static conditions and then mechanically and electrically stimulated in a Bose^®^ bioreactor by uniaxial cyclic stretching for three hours.

The hypotheses of this study were:

1. PVDFhfp/PDMS electrospun fibers impact gene expression of human adipose-derived stem cells with an upregulation of tenogenic and downregulation of pro-inflammatory marker genes in supplement-free basal Dulbecco’s modified eagle medium (DMEM).
2. Aligned PVDFhfp/PDMS electrospun fibers lead to a higher tenogenic induction than corresponding random electrospun fibers [36].
3. Tenogenic gene upregulation is more pronounced after uniaxial cyclic stretching than under mere static cell cultivation [25].

## 2. Materials and Methods

### 2.1. Cells

Human adipose-derived stem cells (ASCs) were isolated from fat tissue with the consent of the patients according to Swiss (KEK-ZH: StV 7-2009) and international ethical guidelines (Clinical Trials.gov Identifier: NCT01218945) as reported in Buschmann et al. [37]. The extraction procedure was performed following Zuk et al. [38]. ASCs were characterized according to established procedures [39,40]. Of the 30 isolated primary ASC lines [37], three ASC lines were randomly selected [41]. The samples for these primary cells had been received from women, aged 54 years (F28, abdominal tissue, BMI: 24.2), 41 years (F15, abdominal tissue, BMI 21.3) and 29 years (F18, liposuction, BMI 24.1), with FXX being the internal number of patient from the comparative study of 30 patients [37]. In addition, human tenocytes that served as positive control were received as a kind gift from Prof. Snedeker, ETH Zurich. Two donors for tenocytes were included in the study; with one donor (patient 485 according to internal numbering): female, age 16 years, cells from *semitendinosius*, passage P3; and the second donor (patient 1062): female, age 19 years, cells isolated from gracilis tendon, not passaged, used as P0.

### 2.2. Multilineage Cell Differentiation

Lineage specific differentiation of ASCs towards the osteogenic, the endothelial, the adipogenic and the chondrogenic cell lineage were achieved using cell culture media supplementation according to Zuk et al. [38]. Von Kossa and Alizarin red staining were used to semi-quantitatively evaluate osteogenic differentiation extent, CD31 immunohistochemical staining to see the endothelial cell differentiation, Alcian Blue staining to evaluate the ability of the ASCs for chondrogenesis and Oil Red O staining to proof adipogenic differentiation—all differentiations have been reported previously [42].

### 2.3. Scaffolds

The amount of 3.5 g poly(vinylidene fluoride-co-hexafluoropropylene) (PVDFhfp, 400,000 Da, Sigma Aldrich, Burlington, MA, USA) was dissolved in 10 mL N,N-dimethylformamide (DMF) to give a 35 *w*/*v*% shell polymer solution [29]. For the PDMS core, PDMS Sylgard^®^ 184 (Dow Corning, Midland, MI, USA) elastomeric base was dissolved in tetrahydrofuran (THF) in a ratio of 10:1 (*w*/*w*).

Electrospun scaffolds were produced at EMPA, St. Gallen, Switzerland. For that purpose, an in-house assembled electrospinning device was used. The process was performed in a temperature and humidity controlled environment, setting the temperature at 30 °C and humidity at 35%. Core–shell PVDFhfp/PDMS meshes, composed of PVDFhfp shell and PDMS core, were prepared by coaxial electrospinning. The polymer solutions for the shell and core were delivered through two different syringes mounted on two different pumps (SP210cZ and Aladdin-1000, WPI, Berlin, Germany) and different flow rates were used for each solution (shell: 18.30 µL min^−1^; core: 3.05 µL min^−1^). Voltage of 10 kV and −1 kV was applied at the cathode and anode, respectively, and the working distance between the spinneret and the collector was 15 cm. The solutions were then electrospun through a coaxial needle (PVDFhfp in the shell and PDMS in the core) either on a plate (random fibers) or on a cylindrical collector 12 cm in diameter (aligned fibers) rotating at 200 rpm. The coaxial needle was characterized by 12 g and 16 g for the outer and inner needle respectively. The surface of the as-prepared scaffolds was investigated by means of scanning electron microscopy (SEM, FEI, Nova NanoSEM 450). Single fibers were analyzed by transmission electron microscopy (TEM, FEI, Philips CM 12, TSS microscopy, New York, NY, USA).

### 2.4. Tissue Engineered Constructs: Cultivation

Patches of 1 cm × 1 cm and 200 ± 23 µm thick PVDFhfp/PDMS squares were incubated in 0.5 mL Dulbecco’s phosphate buffered saline (PBS) (Sigma-Aldrich/Merck & Cie, Schaffhausen, Switzerland) with 100 units penicillin and 100 µg mL^−1^ streptomycin (Sigma-Aldrich/Merck & Cie, Schaffhausen, Switzerland) for 1 h and dried in the laminar flow bench after aspiration of the antibiotic solution. The cell seeding consisted of placing 0.2 × 10^6^ ASCs as a single cell suspension on one side of the scaffolds. The cells were distributed homogenously over the surface. All seeded scaffolds were cultivated in 24-well plates using 0.5 mL Dulbecco’s modified eagle medium (DMEM; Sigma-Aldrich/Merck & Cie, Schaffhausen, Switzerland))with 10% of FBS (Pan Biotech, Aidenbach, Germany), GlutaMAX-I (Sigma-Aldrich/Merck & Cie, Schaffhausen, Switzerland) and penicillin-streptomycin for 1 week in a humidified atmosphere of 95% air and 5% CO_2_ at 37 °C. Medium was changed twice a week. Cell proliferation was determined by alamarBlue™ cell viability assay (ThermoFisher Scientific, Waltham, MA, USA). The alamarBlue™ solution was diluted 1:10 in cell culture medium and incubated for 4 h on the cells. Then, 400 µL were removed and split to four times 100 µL, which were transferred to a 96-well plate. Fluorescence was determined by a Cytation 5 imaging reader (BioTek/Agilent Technologies (Schweiz) AG, Basel, Switzerland, with excitation wavelength of 530 nm and emission wavelength of 590 nm.

### 2.5. Mechanics of Tissue Engineered Constructs

**Figure 1:**
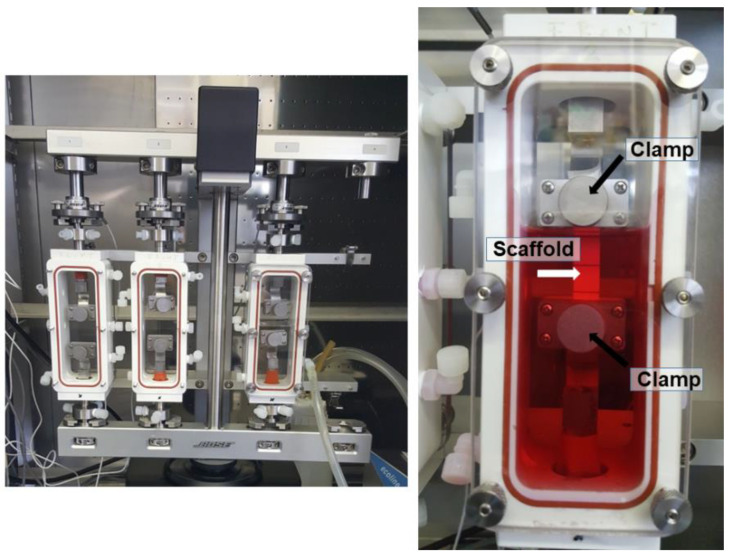
Bose^®^ bioreactor system, with three chambers. Within the chamber, there are two self-made clamps (black arrows) to grip the scaffold material (white arrow). A scaffold attached to the two grips is shown and the chamber is filled with DMEM culture medium (red fluid).

For experiments in the bioreactor, force and displacement were continuously monitored, for cell-seeded scaffolds. Elastic Modulus (E) was assessed. For that purpose, random PVDFhfp/PDMS scaffolds of 7.2 cm × 1.4 cm were seeded with 0.2 × 10^6^ ASCs within a square of approximately 1 cm × 1 cm field in the center and cultivated for one week. Then, they were placed in a bioreactor system (Bose^®^ Electro Force^®^ 5210 Test Instrument, Electro Force Systems Group, Sissach, Switzerland), fixed with in-house made grips (Figure 1). We applied a pre-load of 3 N to have initial tension and then “tared” the load cell before starting the experiment. After a tensile stretching regimen for 3 h, with 1 Hz and 10% strain, the scaffolds were carefully removed and used for quantitative real-time polymerase-chain reaction (RT-qPCR). Sample size was *n* = 3.

In addition, cell-free random scaffolds were tested in air (dry) and in basal DMEM medium (wet). In dry conditions, the scaffolds underwent quasi-static loading with a stretching consisting in 2 cycles (2 ramps) at 0.12 mm/s in displacement control. As for the wet conditions, the scaffolds underwent dynamic cyclic loading consisting in one million cycles. Elastic modulus was calculated on the stress/strain curves after 1 cycle, 100 kcycles and 500 kcycles. Stress was calculated by dividing the load to the cross-sectional area that was for all samples approximately 0.2 mm^2^. SEM pictures of random and aligned fibres were taken on previously gold coated samples (10 nm thickness) at 2 kV voltage. Alignment was assessed on SEM pictures and histograms proving directionality in aligned fibres were extrapolated using the ImageJ software, using the OrientationJ plug-in. Pore size was determined based on SEM pictures; and porosity was assessed according to P = (1 − *ρ*/*ρ* (0)) × 100%, where *ρ* is the density of the scaffold and *ρ*(0) is the density of PVDFhfp (1.78 g/cm^3^). In order to assess the formation of core–sheath fibres, electrospun meshes were embedded in PDMS and cross-sections were cut after immersion in liquid nitrogen. The cold temperature achieved after immersion allowed a clear cut of the sections. Afterwards, the embedded fibres were gold coated and underwent SEM imaging at 2 kV.

### 2.6. Gene Expression

Total RNA was extracted from the electrospun meshes using RNeasy Mini Kit (Qiagen, Hombrechtikon, Switzerland) according to the manufacturer’s instruction. The RNA was quantified using Nanodrop ND-1000 Spectrophotometer (Witec AG, Pfäffikon, Switzerland). An amount of 250 ng RNA was reverse transcribed into cDNA using oligo (dT) 12-18 primer (Invitrogen/ThermoFisher Scientific, Waltham, MA, USA), dNTP mix (Invitrogen/ThermoFisher Scientific, Waltham, MA, USA), DTT (Invitrogen/ThermoFisher Scientific, Waltham, MA, USA), 5× first-strand buffer (Invitrogen/ThermoFisher Scientific, Waltham, MA, USA), RNase inhibitor (Applied Biosystem/ThermoFisher Scientific, Waltham, MA, USA) and SuperScript III reverse transcriptase (Invitrogen/ThermoFisher Scientific, Waltham, MA, USA). RT-qPCR was performed using Fast SYBR^®^ Green master mix (Applied Biosystems/ThermoFisher Scientific, Waltham, MA, USA) as well as primers synthesized by Microsynth (Balgach, Switzerland). For primer sequences, see Table 1. Primers for tenomodulin (*TNMD*), Scleraxis (*SCX*), Tenascin-C (*TNC*), Mohawk (*MKX*), Collagen I (*COL1A1*), Collagen III (*COL3A1*), matrix metalloproteases *MMP-2*, *MMP-9*, alpha-smooth muscle actin (*α-SMA*), interleukin-6 (*IL-6*), *IL-8*, tumor necrosis factor-alpha (*TNF-α*) and protease-activated receptor-2 (*PAR-2*), all human, were used. The number of amplification cycles was 40.

### 2.7. Statistics

All data were analyzed with Microsoft Excel and StatView 5.0.1 softwares. Provided that data were normally distributed (Shapiro–Wilk test) and given variance homogeneity (Levene’s test), parametric one-way ANOVA (analysis of variances) was conducted to compare biomechanics and inductions of gene expression of different experimental conditions. Paired t test was used for elastic modulus E of cell-seeded constructs. *p* values < 0.05 were considered significant and denoted with (*); for *p* < 0.01 (**) and for *p* < 0.001 (***) was used. Values were expressed as median with interquartile range. Plots were made with GraphPad Prism 8.0.0. (224) software.

## 3. Results

### 3.1. PVDFhfp/PDMS Electrospun Membranes

**Figure 2:**
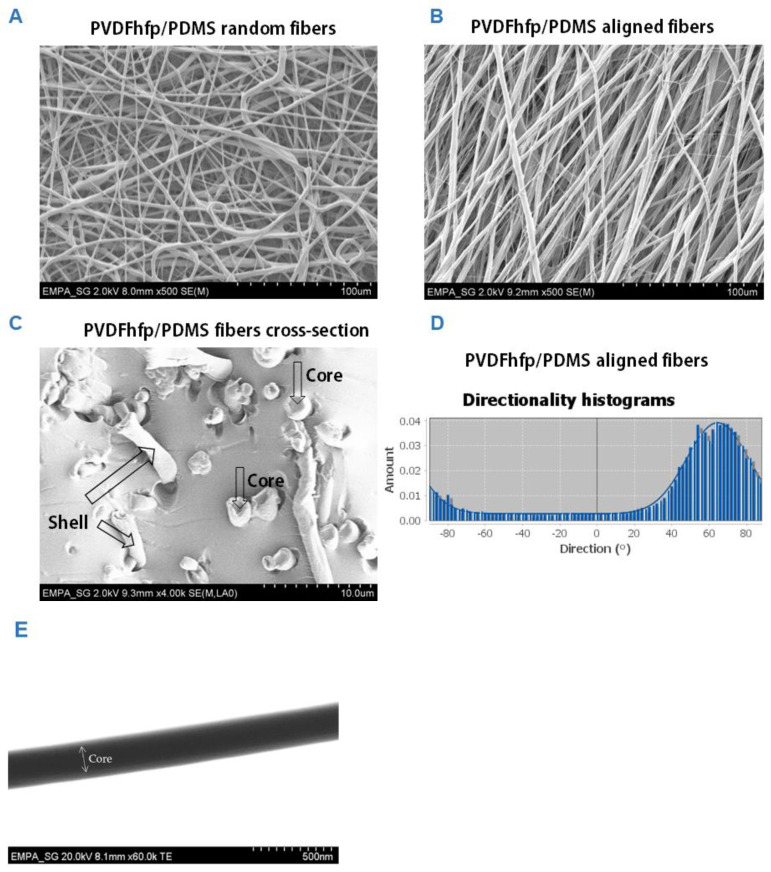
Scanning electron microscopy images (SEM) of random PVDFhfp/PDMS fiber mesh (**A**) and aligned fiber mesh (**B**); cross-section of core–shell fibers, with cores and shells depicted by arrows (**C**) and angles of directionality for aligned fibers (**D**). The fiber diameters were 32.0 ± 10.9 μm, while they were 33.0 ± 10.6 μm for the random and aligned fibers, respectively. TEM picture of core–shell structure (**E**). A white arrow with two arrowheads marks the core. The shell can be seen as a slightly brighter grey border.

Random and aligned fiber meshes were fabricated by coaxial electrospinning (Figure 2). Both random as well as aligned fibers did not show any defects or blobs, nor beads-on-string structure. In contrast, fibers with homogeneous diameter and smooth surface were produced. The diameters in random fiber meshes were 32.0 ± 10.9 μm, while they were 33.0 ± 10.6 μm in aligned fiber meshes. The pore size in random meshes was 170.7 ± 55.3 µm and 147.9 ± 67.6 µm in the aligned fiber meshes. Porosity was 79.3 ± 9.12% for random meshes; and for the aligned fiber meshes it was 84.5 ± 9.71%. For aligned fibers, directionality of the fibers was homogeneous. Under high magnification, the core/shell structure of selected fibers was confirmed (Figure 2C) and in TEM images, the thin sheath of PVDFhfp was observed in contrast to the dark PDMS core (Figure 2E).

### 3.2. Random Versus Aligned Scaffolds; Impact on Gene Expression under Static Conditions

**Figure 3:**
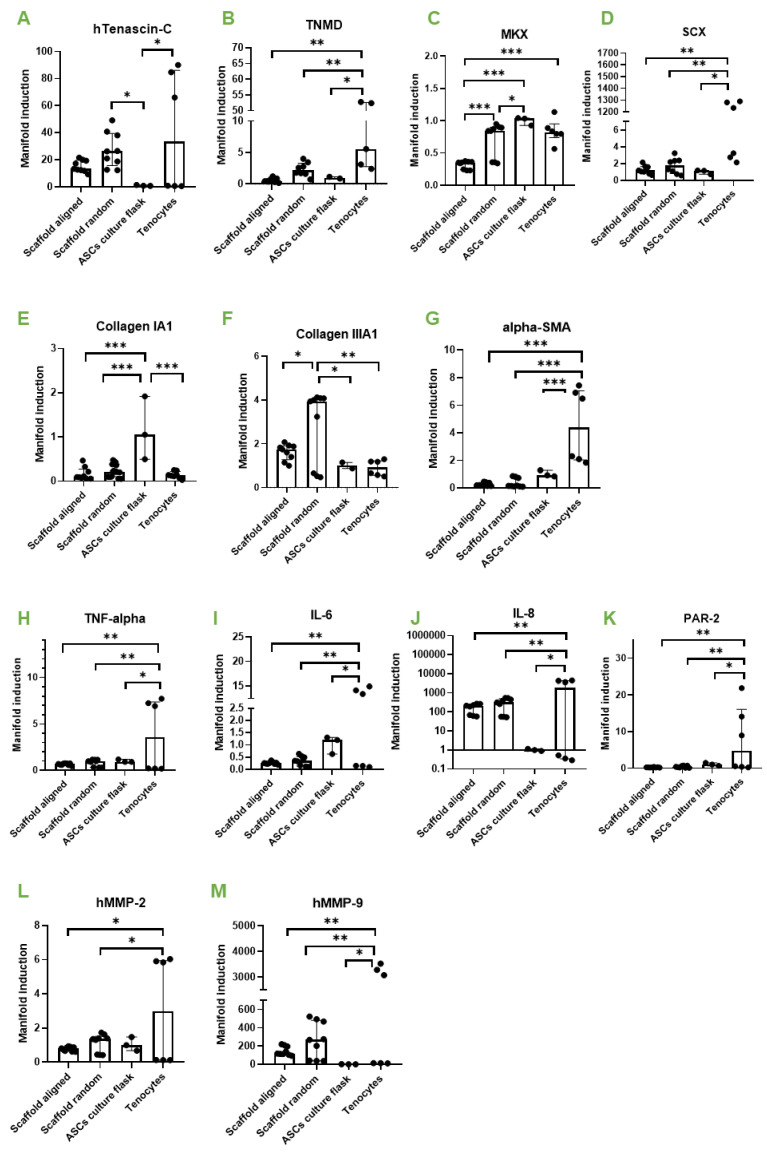
Aligned versus random. Gene expression of human ASCs (hASCs) on aligned PVDFhfp/PDMS, on random PVDFhfp/PDMS, cultivated in normal culture flasks (ASCs in culture flask) and human tenocytes (Tenocytes). Gene expression is shown as manifold induction compared to the ASCs in culture flask group (reference group set to 1). Median and interquartile range are shown. Key: * (*p* < 0.05), ** (*p* < 0.01) and *** (*p* < 0.001) in a one-way ANOVA. (**A**) *TNC*, (**B**) *TNMD*, (**C**) *MKX*. (**D**) *SCX*. (**E**) *Collagen 1A1*. (**F**) *Collagen IIIA1*. (**G**) *α-SMA*. (**H**) *TNF-α*. (**I**) *IL-6*. (**J**) *IL-8*. (**K**) *PAR-2*. (**L**) *MMP-2* and (**M**) *MMP-9*.

Although cells did not proliferate well when seeded on random or on aligned fiber meshes, nevertheless, approximately 3/4 of initial cell numbers were alive after 1 week of static cultivation, which was enough to get insight into the effects on gene expression of ASCs in contact with the materials. The experimental design is shown in SI Figure S1 and cell distribution after 1 week of cultivation is shown in SI Figure S2. As a first step, gene expression for tenogenic and inflammatory markers was analyzed in ASCs on either aligned or random PVDFhfp/PDMS (Figure 3).

The comparison of random and aligned fiber meshes revealed a slight upregulation for typical tendon markers on random scaffolds, such as Tenascin-C (*TNC*) (Figure 3A), Tenomdolin *TNMD* (Figure 3B) and Scleraxis *SCX* (Figure 3D), although there was no statistical significance. Moreover, Mohawk (*MKX*) was significantly enhanced in ASCs on random fibers (Figure 3C). ECM markers were not affected, except collagen III (Figure 3F), which was significantly higher expressed in ASCs on random fibers compared to aligned fibers.

Inflammatory marker genes showed approximately the same expression when random and aligned fibers were compared. However, when cells on scaffolds were compared to tenocytes, they showed a significantly lower expression of *TNF-α, IL-6, IL-8* and *PAR-2* for both, random and aligned fibers (Figure 3H–K). In addition, remodeling markers *MMP-2* and *MMP-9* were significantly lower expressed than in tenocytes (Figure 3L,M). Human tenocytes of two donors used as positive control showed considerable inter-donor variability.

Because the rationale of the study was to generate a novel scaffold material that supports tenogenic differentiation when applied as a wrap around a sutured (flexor) tendon, we decided to further investigate the random fiber meshes under dynamic conditions (cyclic stretching), because they showed more promising results with respect to ASC tenogenic commitment than the aligned fibers (under static cultivation, Figure 3).

### 3.3. Mechanical Properties of Tissue-Engineered Constructs

**Figure 4:**
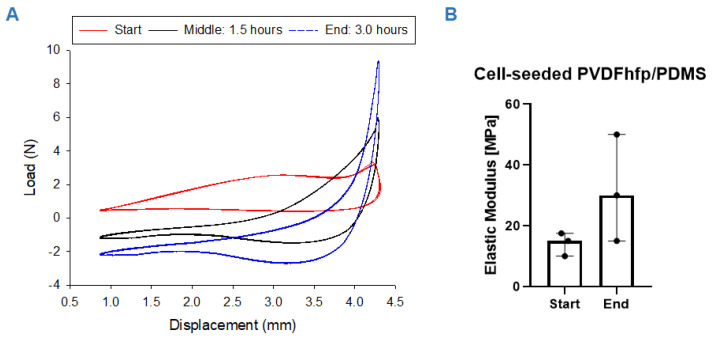
Mechanical properties: Load elongation curves for hASC cell-seeded PVDFhfp/PDMS scaffolds during the 3-h stretching regimen; at the beginning (start: 0 h) (**A**, red curve), in the middle of the experiment (middle: 1.5 h. 5400 cycles) (**A**, black curve) and at the end (end: 3.0 h, 10,800 cycles) (**A**, blue curve). Elastic modulus increased during the experiment, with *p* = 0.25 for *n* = 3 (**B**). Paired t test was used to analyze the elastic modulus data; data are shown as median and interquartile range.

Wet cell-seeded scaffolds were cultivated for three hours under a stretching regimen of 10% strain under displacement control. During this period, E modulus increased gradually from 14.2 ± 3.8 MPa to 31.7 ± 17.6 MPa (*n* = 3, for paired *t* test *p* = 0.2495). The large standard deviation found at the endpoint of the stretching experiments (17.6 MPa) reflects the different responsiveness of the biological samples to mechanical/electrical stimulation. Figure 4 shows typical load elongation curves at the beginning (0 cycles), after 1.5 h (5400 cycles) and at 3 h (10,800 cycles) of cyclic stretching. As we applied a pre-load and due to relaxation of samples and re-orientation of fibers because of the dynamic loading, the load appeared to be negative.

For comparison of the mechanical properties of the cell-seeded constructs with cell-free scaffolds, elastic modulus and residual strain for the cell-free situation of the random fiber meshes are reported in the Supporting Information (SI Figure S3).

The elastic modulus and the residual strain of cell-free random PVDFhfp-PDMS electrospun core–shell fiber meshes was assessed by quasi-static loading (SI Figure S2A) in dry conditions, amounting to 3.3 ± 1.0 MPa. After the first cycle of loading, the elastic modulus showed comparable value featuring a non-significant reduction to 2.6 ± 1.2MPa (SI Figure S2C). Electrospun membranes were also affected by deformation, resulting in a residual strain of 27 ± 1.3% after the first loading cycle in dry conditions and a significantly lower residual strain of 16 ± 3.1% after the second cycle (SI Figure S2F).

Under wet conditions realized in culture medium DMEM and dynamic loading, random PVDFhfp/PDMS meshes showed a progressive decrease in the stress until stabilization after 200 kcycles (SI Figure S2B). Elastic modulus exhibited values amounting to 11.8 ± 9.9 MPa and 5.4 ± 3.2 MPa after the first and 500 loading kcycles, respectively (SI Figure S2D). The residual strain under wet conditions significantly decreased from 27 ± 2.6% to 15 ± 1.9% and to 4.3 ± 1.7% for 1 cycle, 100 kcycles and 500 kcycles, respectively (SI Figure S2F).

### 3.4. Dynamic Compared to Static Cultivation

**Figure 5:**
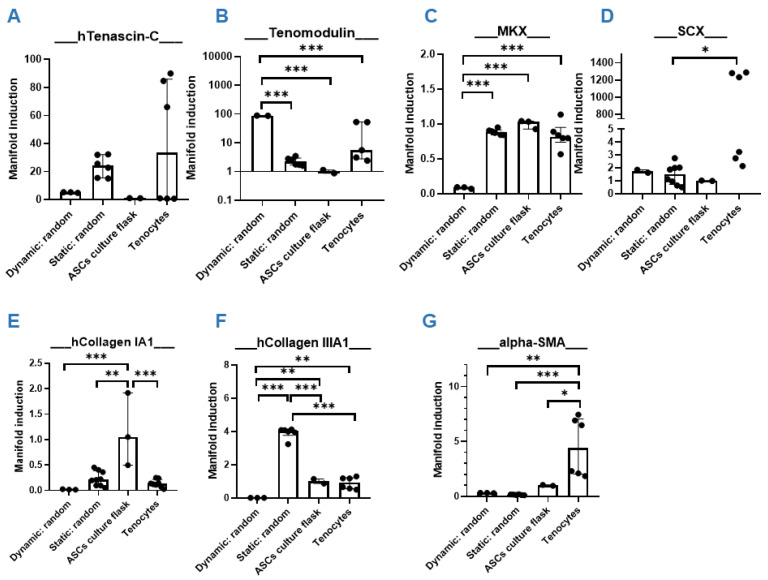

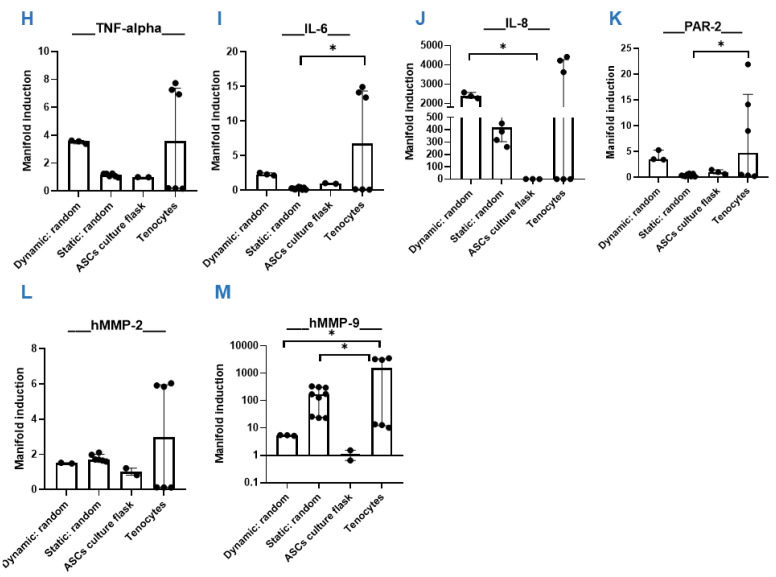
Dynamic versus static. Quantitative RT-PCR results of hASC on PVDFhfp/PDMS random fiber mesh under static and dynamic conditions. Gene expression (=manifold induction) of ASCs under dynamic conditions, static conditions or in culture flask (reference set to 1) or of human tenocytes as control was compared (**A**–**M**). Statistical analysis was performed with one-way ANOVA and data are shown as median and interquartile range, with * (*p* < 0.05), ** (*p* < 0.01) and *** (*p* < 0.001).

In order to determine if a dynamic cultivation step would add benefit to the intended tenogenic commitment of ASCs seeded on random PVDFhfp/PDMS scaffolds, cells were cultivated for 1 week under normal static culture conditions and then cyclically stretched for 3 h to 10% strain at 1 Hz (Figure 5). Significantly higher *TNMD* gene expression was observed under dynamic culture conditions compared to static culture (Figure 5B). Here, values were normalized to ASCs without scaffold and cultured under static conditions (culture flask). However, *MKX* showed the opposite (Figure 5C) as the static cultivation led to a significant increase compared to dynamic cultivation. *TNC* was also upregulated compared to dynamic conditions by trend (Figure 5A).

As for *collagen I*, an increase for static conditions was observed, although not statistically significant (Figure 5E). For the ECM markers, *collagen III* was significantly higher under static compared to dynamic conditions (Figure 5F). Noteworthy is the significant downregulation of the pro-fibrotic marker gene *α-SMA* in scaffold cultures (both dynamic and static cultivation), when compared to tenocytes (Figure 5G).

Although statistically not significant, all four pro-inflammatory markers *TNF-α, IL-6, IL-8* and *PAR-2* were increased under the dynamic stretching regimen compared to static cultivation (Figure 5H–K). Matrix metalloproteases were not significantly impacted through dynamic stretching compared to static conditions.

### 3.5. Static Cultivation on Random PVDFhfp/PDMS—The Favorite

**Figure 6:**
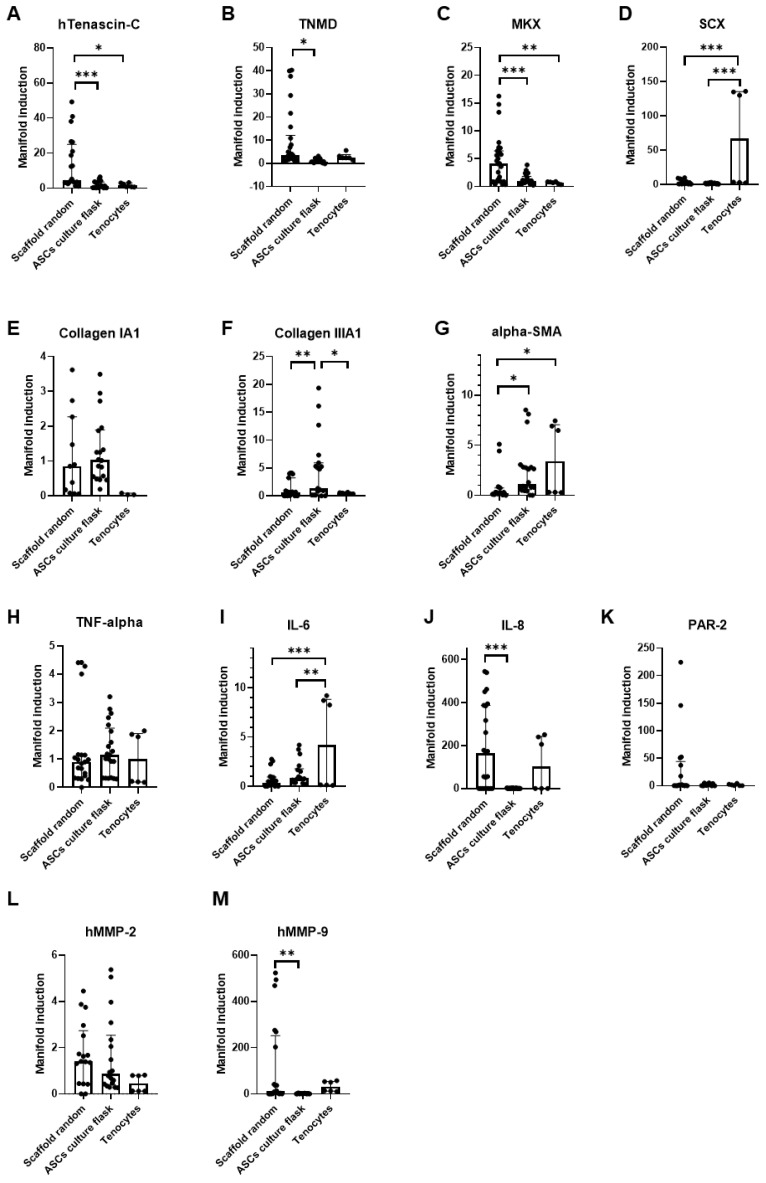
Random PVDFhfp/PDMS under static conditions. Quantitative RT-PCR results of 3 human ASC donors. Statistical analysis was performed with one-way ANOVA and data are shown as median and interquartile range, with * (*p* < 0.05), ** (*p* < 0.01) and *** (*p* < 0.001). (**A**) *TNC*, (**B**) *TNMD*, (**C**) *MKX*. (**D**) *SCX*. (**E**) *Collagen 1A1*. (**F**) *Collagen IIIA1*. (**G**) *α-SMA*. (**H**) *TNF-α*. (**I**) *IL-6*. (**J**) *IL-8*. (**K**) *PAR-2*. (**L**) *MMP-2* and (**M**) *MMP-9*.

As the favorite condition when static and dynamic cultivation were compared (Figure 5), ASCs of three human donors were finally cultured on random PVDFhfp/PDMS meshes under static conditions for one week and compared to their ASCs in culture flask (Figure 6). A significant upregulation of tendon markers *TNC, TNMD* and *MKX* was found (Figure 6A–C), when ASCs were in contact with PVDFhfp/PDMS random fibers compared to ASCs in culture flask. *Collagen III* and *α-SMA* gene expression were significantly lower than for ASCs in culture flask (Figure 6F,G).

Inflammatory marker genes did not differ, comparing ASCs seeded on random PVDFhfp/PDMS and culture flask. The only exception is *IL-8*, which was significantly enhanced on the scaffold (Figure 6J). From the two matrix metalloproteases analyzed, MMP-9 was significantly higher expressed in ASCs seeded on the random scaffold compared to the culture flask (Figure 6M).

## 4. Discussion

In order to support tendon healing and minimize adhesion to neighbor tissue, smart functional materials are needed, providing ideal cues for tenogenic differentiation and modulating inflammatory response [43] towards a scar-less regenerative healing [44]. Physical membrane barriers around sutured tendons in form of thin electrospun fiber meshes [7] might be an option to address both aims, i.e., reduce adhesion and support tenogenesis. Our current study presents a novel hydrophobic coaxially electrospun fiber mesh, based on PVDFhfp/PDMS, featuring a PVDFhfp shell and a PDMS core. Human ASCs were seeded on either aligned or random fibers, and a dynamic cultivation step was tested, resulting in mechanical stretching and simultaneous electrical stimulation of the stem cells due to piezoelectric PVDFhfp. Gene expression was used as readout.

Cultivation on random fiber meshes increased Mohawk (*MKX*) gene expression significantly compared with aligned fiber meshes, as well as Tenascin-C (*TNC*) and Scleraxis (*SCX*) by trend (Figure 3). Cell-seeded random scaffolds showed a slight increase in stress after 10 kcycles (Figure 4) which was instead not observed in common stress/strain curves associated to cell- free PVDFhfp/PDMS meshes (SI Figure S2). When cell-seeded random scaffolds were exposed to cyclic uniaxial stretching for 3 h and compared to corresponding static culture, *MKX* was significantly downregulated, while tenomodulin (*TNMD*) was significantly upregulated; and inflammatory markers, such as *PAR-2, TNF-α, IL-6* and *IL-8*, showed a slight increase (Figure 5). Finally, static cultivation of random scaffolds seeded with ASCs of three donors confirmed the significant increase in tenogenic markers *TNC, TNMD* and *MKX* compared to ASCs in culture flask, while *collagen III* and *α-SMA* were significantly downregulated (Figure 6).

Although tenogenic differentiation of stem cells can be achieved by supplementing the culture medium with growth and differentiation factor-5 (GDF-5 = bone morphogenetic protein-14, BMP-14) [4,6,45], our aim was to direct ASCs commitment towards a tenogenic phenotype via contact to the PVDFhfp surface of the fibers, without further induction medium, i.e., no chemical supplementation.

There are many reports on the comparison of random versus aligned electrospun fibers, serving as scaffold materials for stem cells [46,47,48]. For example, osteogenic differentiation of BMSCs was found to be favored on aligned nanofibers made of poly-L-lactic acid (PLLA) compared with random nanofibers [49]. Moreover, a study with human apical papilla cells for dental pulp regeneration showed that seeding these cells on aligned nanofibers of ε-caprolactone led to higher cell migration, viability, proliferation, adhesion and spreading, as well as collagen synthesis, compared to random fibers [50]. As for tenogenic differentiation, reports are controversial. Although a study shows aligned fibers to lead to improved tenogenesis compared with random fibers [51], another study employing human ASCs on random and aligned PLLA fibers, with additional immobilized PDGF-BB, resulted in nanofiber alignment having no effect [52]. Surprisingly, the gene expression resulting for human ASCs seeded on random PVDFhfp/PDMS showed higher *TNMD* (~4 fold), *TNC* (~2fold) and *MKX* expression (~2fold) compared to corresponding aligned fibers (Figure 3). This indicates that not only the topography, but also the chemical composition at the interface to the cells plays an important role—with the PVDFhfp surface in contact to the cells, leading to a more tenogenic commitment when the cells were on random fibers. PVDFhfp is very hydrophobic, making particularly interesting the finding of increased *TNC* gene expression. As TNC is an ECM stress protein, its main function consists in the modulation of cell adhesion through binding to fibronectin III [53]. Here, *TNC* gene expression was increased in random fibers compared to aligned fibers. On random fibers, ASCs are supposed to attach less easily to the mesh compared to aligned fibers [54], so that *TNC* is obviously increased to counteract the difficult microenvironment and to support further adhesion on random fibers (Figure 3). Of note, this upregulation of *TNC* was even more pronounced when compared to ASCs in the culture flask (~25fold).

In addition to the *TNC* increase, *MKX* was significantly higher when ASCs were on random than aligned fibers. The transcription factor MKX is a central factor regulating tendon-related gene expression; an overexpression of MKX was reported to elevate tendon-related markers in MSCs [55] and MKX knockout models were shown to exhibit heterotopic ossification of the Achilles tendon in Mkx(−/−) mice and rats [56]. Given these facts, a prominent induction of in ASCs that are in contact with random PVDFhfp/PDMS holds great promise to act beneficially with regard to induce tenogenic commitment, e.g., for mesenchymal stem cells present in the synovial fluid [35,57].

When cells on random PVDFhfp/PDMS were cultivated statically for one week and then subjected to a 3-h tensile cyclic stretching at 10% strain, *TNMD* gene expression significantly increased (40 fold compared to corresponding static cultivation; 100 fold compared to ASC in culture flask without scaffold, Figure 5, step 2 in SI Figure S1). TNMD is found primarily in dense hypo-vascular connective tissue; particularly in tendons, skeletal muscle and ligaments [58]. It is necessary for tenocyte proliferation and collagen type I fibril maturation [59]. In addition, TNMD is mechanosensitive (C-terminus co-localized with collagen I fiber in the ECM) and is required for a correct collagen I fibril adaption to mechanical load. On this regard, previous in vivo studies performed on a mice model showed that TNMD knockout to limit mice performance during endurance running [60]. Hence, our finding of an obvious increase of *TNMD* in ASCs seeded on PVDFhfp/PDMS can be judged beneficial with regard to stem cell commitment towards the tenogenic phenotype. The seeding of ASCs on random PVDFhfp/PDMS furthermore leads to a significant reduction in the profibrotic marker *α-SMA* compared to tenocytes (Figure 5), for both static and dynamic cultivation. As a consequence, the lowered *α-SMA* expression goes hand in hand with the high *TNMD* gene expression—because TNMD is reported to prevent fibrovascular scar formation during early tendon healing [61].

In contrast, the crucial and central transcription factor *MKX* was significantly higher under static conditions compared to dynamic cultivation, which has to be judged even more important than the increase of *TNMD* under dynamic conditions, because in addition to *MKX* also *TNC* was upregulated under static conditions. Additionally, ECM markers *collagen III* and *I* were increased under static conditions.

A further important finding and coherent with literature was the upregulation of inflammatory marker genes through exercise or tensile stretching [62]. We found a slight, but not statistically significant increase of *TNF-α, IL-6, IL-8* and *PAR-2* under dynamic conditions (Figure 5). In vivo studies have shown that mechanical loading of tendons leads to higher collagen I synthesis, dependent on auto- and paracrine action through growth factors, such as TGF-β, IGF-1 and also IL-6. During exercise, *IL-6* is increased and it is reported that it may act as a mediator of loading induced collagen I synthesis [63]. However, in our experiments, *collagen I* gene expression was reduced under stretching, going along with downregulation of *collagen III*.

Taken together, the pronounced increase in inflammatory markers as well as the decreased ECM markers under dynamic conditions make the additional cultivation step in the bioreactor less promising than first expected. To keep things simple during preparation of a suitable wrap to be implanted around tendons, static cultivation of stem cells on PVDFhfp/PDMS was overall judged to be the favorite over dynamic cultivation.

Dynamic cultivation of cell-seeded PVDFhfp/PDMS meshes led to an increase of the E modulus over a period of 3 h during cyclic uniaxial tensile stretching (Figure 4). This finding stands in contrast to the cell-free situation where the E modulus decreased over repeated cyclic stretching (SI Figure S2) because the fibers of the material adapted to the stretching regimen, first by aligning toward the direction of the stretching and then following the well-known viscoelastic behavior (stress relaxation) of polymers [64]. In the cell-seeded condition, either the pores of the fiber mesh got filled with deposited ECM components of the highly stimulated cells (mechanical stretch and electrical stimulus by the piezoelectric characteristics [15]), or the surface of the fiber mesh conglutinates so that the culture medium cannot intrude easily into the pores anymore. Previous studies have shown that a very short time of dynamic loading with only 15 min had significantly affected cells seeded on scaffolds [65]. It has to be considered that the cell-seeded constructs were cultivated statically for 1 week before dynamic activation and our results from the static culture indicate a pronounced increase in *TNMD* gene expression compared to ASCs in culture flasks (Figure 5B). This might therefore have led to some matrix deposition within the pores of the scaffolds during static culture, resulting in a pore-closing effect during subsequent dynamic stimulation—with the curve resembling a typical elastic polymer (after 10,800 cycles).

Based on the mechanical and PCR findings, as a final step (SI Figure S1), we seeded ASCs of three different human donors on random PVDFhfp/PDMS and compared the gene expression with the corresponding ASCs in the culture flasks under static cultivation (Figure 6). Although there was some inter-donor variability, average upregulation of *MKX, TNC* and *TNMD* was significant when cells were in contact with the surface PVDFhfp of the fibers. Notably, pro-fibrotic *α-SMA* was significantly downregulated as well, paving the way for an accurate support of tendon healing [44], in case cell-seeded PVDFhfp/PDMS membranes are wrapped around a lacerated and sutured tendon.

## 5. Conclusions

We present a novel coaxially electrospun core–shell PVDFhfp/PDMS scaffold material, which is highly elastic and can potentially be used as a wrap around surgically sutured tendons after repair. This scaffold can be used as an anti-adhesion barrier. Interactions with stem cells in the sense of a tenogenic commitment have been shown to be more prominent when random fibers are used compared to aligned fibers. Although *TNMD* gene expression is significantly upregulated in ASCs on PVDFhfp/PDMS fibers after a 3-h cyclic stretching regimen to 10%, other important genes for tenogenesis, such *as collagen I* and *III* as well as the central transcription factor *MKX* experienced a downregulation. We conclude that a random PVDFhfp/PDMS wrap might lead to better results if only static cultivation is used before the cell-loaded construct is implanted, because dynamic stretching induced also a non-negligible upregulation of several inflammatory marker genes. As an alternative, random PVDFhfp/PDMS could also be used without cells, influencing mesenchymal stem cells present in the synovium of intrasynovial tendons [35] towards tenogenic commitment, while inflammatory reaction will be kept in check.

## Figures and Tables

**Table 1:** Primer sequences (h = human) used for reverse transcription-polymerase chain reaction gene expression analysis.

Genes	5′-3′	Primers
*hTenascin-C*	Forward	GGTGGATGGATTGTGTTCCTGAGA
	Reverse	CTGTGTCCTTGTCAAAGGTGGAGA
*hMMP-2*	Forward	TGCGACCACAGCCAACTACG
	Reverse	TGGGACAGACGGAAGTTCTTGG
*hMMP-9*	Forward	GACGCCGCTCACCTTCACTC
	Reverse	TTGGAACCACGACGCCCTTG
*hCOL3A1*	Forward	CAGCGGTTCTCCAGGCAAGG
	Reverse	CTCCAGTGATCCCAGCAATCCC
*hCOL1A1*	Forward	TGA CGA GAC CAA GAA CTG
	Reverse	CCA TCC AAA CCA CTG AAA CC
*hTNMD*	Forward	CCATGCTGGATGAGAGAGGTT
	Reverse	TTGGTAGCAGTATGGATATGGGT
*hTNF-α*	Forward	CGGACACCATGGACAAGTTT
	Reverse	GAAAGCCTTGCAGAGGTCAG
*hα-SMA*	Forward	ACTGAGCGTGGCTATTCCTCCGTT
	Reverse	GCAGTGGCCATCTCATTTTCA
*hMKX*	Forward	TCAAGGACAACCTCGGCCTG
	Reverse	ACGGGTTGTCACGGTGCTTG
*hSCX*	Forward	AGAACACCCAGCCCAAACAG
	Reverse	GGCCACCTCCTAACTGCGAATC
*hIL-6*	Forward	GTAGCCGCCCACACAGACAGCC
	Reverse	GCCATCTTTGGAAGGTTC
*hIL-8*	Forward	TCTGCAGCTCTGTGTGAAGGT
	Reverse	TGAATTCTCAGCCCTCTTCAA
*hPAR2*	Forward	GTTGATGGCACATCCCACGTC
	Reverse	GTACAGGGCATAGACATGGC
*gapdH*	Forward	ACCACAGTCCATGCCATCAC
	Reverse	TCCACCACCCTGTTGCTGTA

## Data Availability

Data available on request due to restrictions privacy. The data presented in this study are available on request from the corresponding author.

## Supplementary Materials

The following are available online at https://www.mdpi.com/article/10.3390/bioengineering9010021/s1, Figure S1: Experimental step-by-step design for cell culture experiments; first row: adipose-derived stem cells (ASCs) were cultured in culture flask or seeded onto aligned or random fiber meshes of PVDFhfp/PDMS scaffolds; as positive control, human tenocytes were cultured in culture flasks. All experiments were done under static conditions. Second row: the most promising condition (static, random) was then compared to ASCs seeded on random fibers, but cultivated under dynamic conditions (i.e., stretching) in a Bose^®^ bioreactor. Third row: the better condition with respect to tenogenic commitment of step 2 (static, random) was chosen and then performed for another two human ASC donors, with corresponding ASCs also cultivated in culture flasks as reference. The favorites of each step are marked by boxes, Figure S2: Cell-seeded scaffolds (aligned and random) after 1 week of cultivation, Figure S3: Cell-free situation: Stress–strain curve representative for random PVDFhfp/PDMS electrospun meshes (A) and stress pattern over number of cycles of such meshes during dynamic loading (B). Cell-free core–shell PVDFhfp/PDMS meshes achieved full relaxation after 200 kcycles and featured a constant trend under dynamic loading in wet conditions until 500 kcycles. Elastic Modulus was measured in air (C) and in culture medium DMEM (D), residual strain measured in air (E) and in culture medium (F). Median and interquartile range are shown. Key: * (*p* < 0.05), ** (*p* < 0.01) and *** (*p* < 0.001) in a unpaired *t* test (2 groups) and a one-way ANOVA (3 groups).
