# Supplementary material for: Impact of Electrospun Piezoelectric Core–Shell PVDFhfp/PDMS Mesh on Tenogenic and Inflammatory Gene Expression in Human Adipose-Derived Stem Cells: Comparison of Static Cultivation with Uniaxial Cyclic Tensile Stretching
Source: Bioengineering (Basel). 2022 Jan 8;9(1):21. doi: 10.3390/bioengineering9010021 (PMC8772741; doi:10.3390/bioengineering9010021)
Supplement: Supplementary file 1 [file bioengineering-09-00021-s001.zip › bioengineering-1504216-supplementary.pdf]

Impact of Electrospun Piezoelectric Core–Shell PVDFhfp/PDMS Mesh on Tenogenic and Inflammatory Gene Expression in Human Adipose-Derived Stem Cells: Comparison of Static Cultivation with Uniaxial Cyclic Tensile Stretching

Walter Baumgartner [1], Petra Wolint [1], Silvan Hofmann [1], Cléa Nüesch [1], Maurizio Calcagni [1], Marzia Brunelli [2] and Johanna Buschmann [1,*]

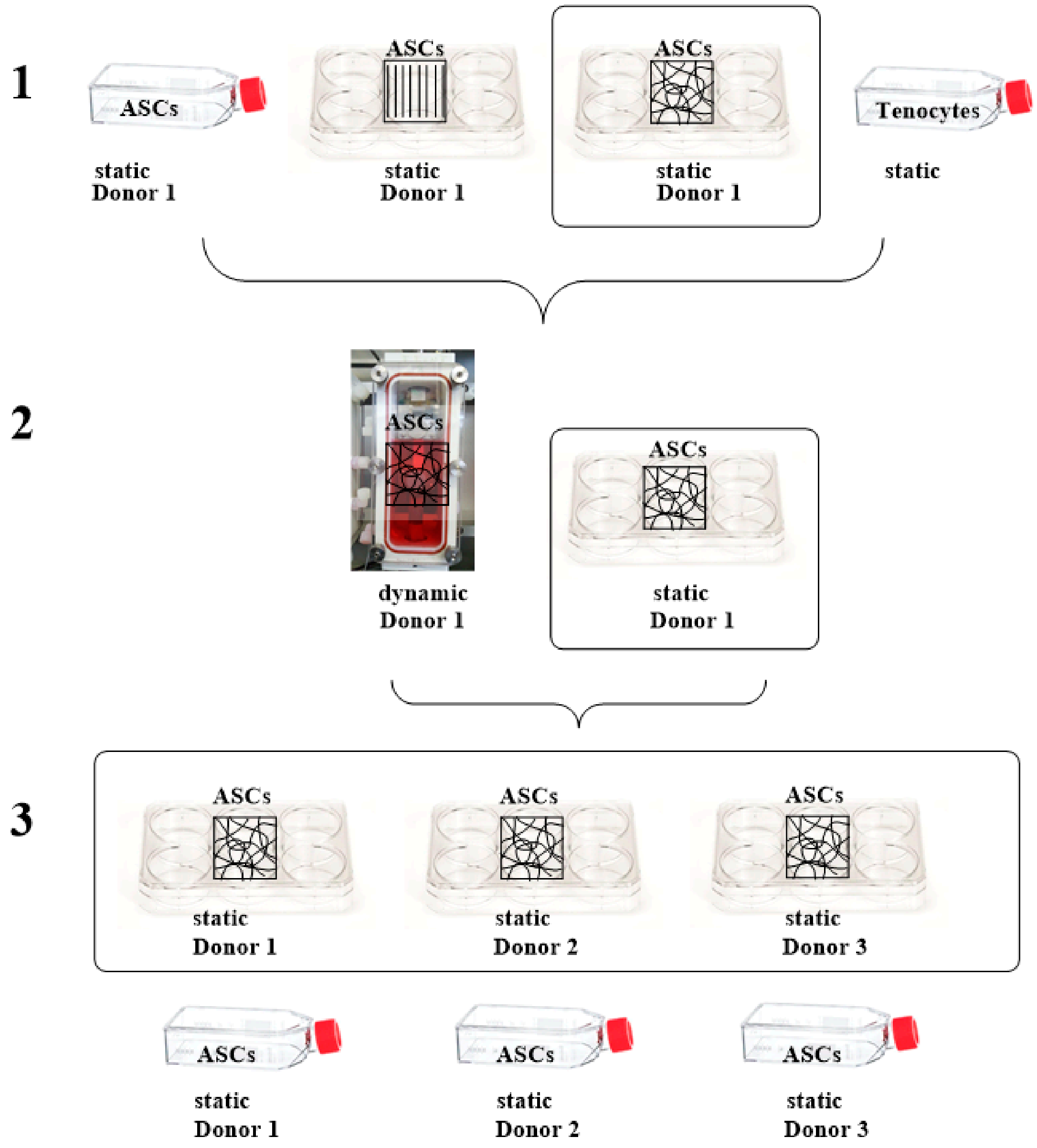

Figure S1. Experimental step-by-step design for cell culture experiments; first row: adipose-derived stem cells (ASCs) were cultured in culture flask or seeded onto aligned or random fiber meshes of PVDFhfp/PDMS scaffolds; as positive control, human tenocytes were cultured in culture flasks. All experiments were done under static conditions. Second row: the most promising condition (static, random) was then compared to ASCs seeded on random fibers, but cultivated under dynamic conditions (i.e. stretching) in a Bose® bioreactor. Third row: the better condition with respect to tenogenic commitment of step 2 (static, random) was chosen and then performed for another two human ASC donors, with corresponding ASCs also cultivated in culture flasks as reference. The favourites of each step are marked by boxes.

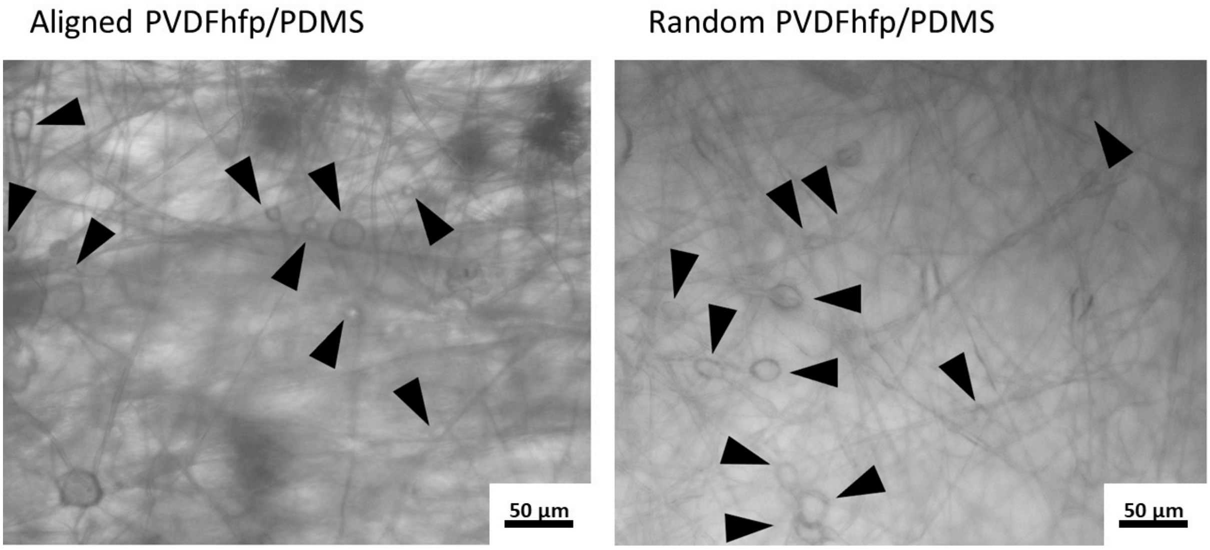

Figure S2. ASCs on aligned and random fibre meshes after 1 week of static cultivation. Arrow heads point to selected cells.

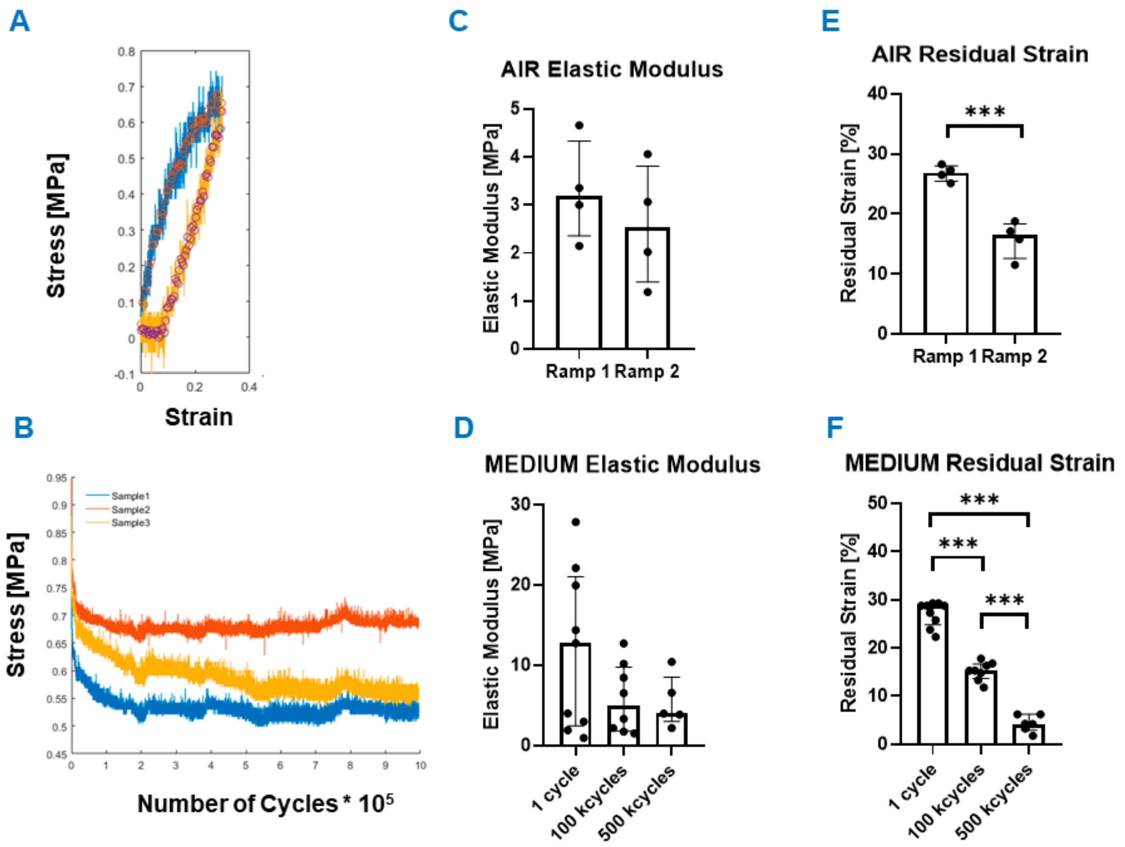

Figure S3. Cell-free situation: Stress-strain curve representative for random PVDFhfp/PDMS electrospun meshes (**A**) of a single representative sample during dynamic loading (**B**). The blue line represents the first cycle of loading. The yellow line represents the second loading cycle, underlying the relaxation occurring due to fibers re-orientation toward the direction of loading. The orange dots represent the curve resulting from the mathematical model used for calculation of E modulus according to the formula (stress= E*strain).(**B**) Stress pattern over number of cycles for 3 tested samples (sample1= orange curve, sample2=yellow curve, sample3=blue curve). Cell-free core-shell PVDFhfp/PDMS meshes achieved full relaxation after 200 kcycles and featured a constant trend under dynamic loading in wet conditions until 500 kcycles. Elastic Modulus was measured in air (**C**) and in culture medium DMEM (**D**), residual strain measured in air (**E**) and in culture medium (**F**). Median and interquartile range are shown. Key: *** ($p<0.001$) in a unpaired t test (2 groups) and a one-way ANOVA (3 groups).
